# Risk‐based management of invading plant disease

**DOI:** 10.1111/nph.14488

**Published:** 2017-03-28

**Authors:** Samuel R. Hyatt‐Twynam, Stephen Parnell, Richard O. J. H. Stutt, Tim R. Gottwald, Christopher A. Gilligan, Nik J. Cunniffe

**Affiliations:** ^1^ Department of Plant SciencesUniversity of CambridgeDowning StreetCambridgeCB2 3EAUK; ^2^ School of Environment and Life SciencesUniversity of SalfordManchesterM5 4WTUK; ^3^ USDA Agricultural Research Service2001 South Rock RoadFort PierceFL34945USA

**Keywords:** adaptive control, citrus canker, disease management, eradication, risk‐based control, stakeholders, stochastic epidemic model

## Abstract

- Effective control of plant disease remains a key challenge. Eradication attempts often involve removal of host plants within a certain radius of detection, targeting asymptomatic infection. Here we develop and test potentially more effective, epidemiologically motivated, control strategies, using a mathematical model previously fitted to the spread of citrus canker in Florida.
- We test risk‐based control, which preferentially removes hosts expected to cause a high number of infections in the remaining host population. Removals then depend on past patterns of pathogen spread and host removal, which might be nontransparent to affected stakeholders. This motivates a variable radius strategy, which approximates risk‐based control via removal radii that vary by location, but which are fixed in advance of any epidemic.
- Risk‐based control outperforms variable radius control, which in turn outperforms constant radius removal. This result is robust to changes in disease spread parameters and initial patterns of susceptible host plants. However, efficiency degrades if epidemiological parameters are incorrectly characterised.
- Risk‐based control including additional epidemiology can be used to improve disease management, but it requires good prior knowledge for optimal performance. This focuses attention on gaining maximal information from past epidemics, on understanding model transferability between locations and on adaptive management strategies that change over time.

## Abstract 2

See also the Commentary on this article by Vicent & Blasco, **214**: 905–908.

## Introduction

Outbreaks of new and emerging plant diseases threaten food security (Rosegrant & Cline, 2003; Strange & Scott, 2005) and ecosystem services (Boyd *et al*., 2013). Rates of introduction of exotic pathogens have increased in recent years, driven largely by altered patterns and increasing rates of travel and trade (Brasier, 2008). Ongoing high‐profile invasions include citrus canker (caused by *Xanthomonas axonopodis*; Gottwald *et al*., 2002a) and huanglongbing (or citrus greening, caused by *Candidatus* Liberibacter spp.; Gottwald, 2010) in the United States and Brazil (Belasque *et al*., 2010). Other prominent examples include sudden oak death (caused by *Phytophthora ramorum*) in the United States (Rizzo *et al*., 2005) and western Europe (Brasier & Webber, 2010), as well as olive quick decline syndrome in southern Europe (caused by *Xylella fastidiosa*; Martelli, 2016). The food security of sub‐Saharan Africa is threatened by cassava brown streak disease (caused by Cassava brown streak virus; Legg *et al*., 2011), maize lethal necrosis (caused by co‐infection with Maize chlorotic mottle virus and a potyvirus such as Sugarcane mosaic virus; Mahuku *et al*., 2015) and emerging races of wheat stem rust (caused by *Puccinia graminis*) virulent on previously resistant varieties of wheat (Singh *et al*., 2011). The huge impacts of these – and other – new and emerging plant diseases focus our attention on understanding when, where and how invading pathogens can be controlled (Cunniffe *et al*., 2015a, 2016).

Assuming there is sufficient surveillance to ensure epidemics remain small when first detected (Parnell *et al*., 2015), eradication becomes a realistic proposition (Rejmánek & Pitcairn, 2002). Eradication schemes typically involve reactive treatment within a particular distance of infected sites (Parnell *et al*., 2009). The underlying idea is to remove hosts that are likely to be infected, but do not yet show symptoms (Cunniffe *et al*., 2015b). Disease management based on this underlying principle is currently in progress for olive quick decline in Italy (Martelli, 2016) and wheat blast in Bangladesh (Callaway, 2016). It was also the focus of efforts to control sudden oak death in Oregon (Peterson *et al*., 2015) and ramorum disease in the UK (DEFRA, 2014), at least initially. However, as illustrated by the continued spread of these *P. ramorum* epidemics, neither of which can realistically now be eradicated, attempted eradication is often unsuccessful. It can also be extremely damaging; for example, the ongoing attempt to manage the olive quick decline epidemic in Italy involves removal of all host plants with 100 m of detected disease, leading to removal of hundreds of ancient olive trees for every detected tree (Martelli, 2016). The social and economic consequences, in a region of Italy in which this crop is an integral part of the local heritage, are proving to be extremely significant.

A number of factors potentially underlie failures to manage disease. Detection can be difficult and expensive (Parnell *et al*., 2014), and there is often cryptic infection, in which hosts are infectious even before showing detectable symptoms (Fraser *et al*., 2004). Long‐distance spread is routine for certain pathogens (Brown & Hovmoller, 2002), with extensive creation of new disease foci (Wingen *et al*., 2013). The epidemiology of exotic pathogens can be imperfectly characterised (Cunniffe *et al*., 2014), with little known about rates of spread (Neri *et al*., 2014), probabilities of invasion following introduction (Thompson *et al*., 2016b), transmission pathways (Peterson *et al*., 2014) and even host species that can be infected (Brasier & Webber, 2010). Additionally, management programmes sometimes face opposition from affected stakeholders, particularly when hosts without visible symptoms must be treated and/or removed. For agriculturally important hosts, plants are often distributed in a matrix of commercial and residential landscapes, putting commercial concerns and residential homeowners at odds. This can lead to social and legal disputes, which in turn can cause long delays between detection and control (Gottwald, 2007). Often, too, there is simply insufficient budget for eradication to be realistic (Cunniffe *et al*., 2016).

Faced with these challenges, making efficient use of available resources is crucial. However, the most common methodology driving local reactive management, which usually involves treating or removing all hosts within a particular distance of infected sites, is rather unsophisticated. Contact tracing is routine for human pathogens (Fraser *et al*., 2004), as are trace forward and backward surveys for plant pathogens that spread via trade (Hernandez Nopsa *et al*., 2015). Additionally, a range of complex reactive vaccination (Keeling *et al*., 2003; Tildesley *et al*., 2006) and pre‐emptive culling (Kao, 2003; te Beest *et al*., 2011) strategies have been proposed for animal diseases, based on patterns of spread observed before the time of control. Collectively these observations suggest more elaborate control strategies informed by additional epidemiology can be made to be effective. For plant disease, recent work has used mathematical modelling to test how large regions within a spreading epidemic can be prioritised relative to each other for surveillance (Sutrave *et al*., 2012; Parnell *et al*., 2014) as well as for treatment (Cunniffe *et al*., 2016). We also have a very good understanding of factors promoting success of constant radius control at small scales (Cook *et al*., 2008; Dybiec *et al*., 2009; Parnell *et al*., 2009, 2010; Cunniffe *et al*., 2015b). However, how local control of plant disease around a newly detected focus can be improved by including additional epidemiological insight is yet to be investigated, even though repeated local control underpins any attempt to eradicate.

We therefore investigate epidemiologically motivated management strategies to locally eradicate an isolated outbreak of a newly invading plant disease. We test the strategies using a spatially explicit, individual‐based, stochastic, compartmental epidemic model, previously parameterised for the spread of citrus canker (caused by the bacterium *X. axonopodis*) in urban tree populations in Florida (Cook *et al*., 2008; Parnell *et al*., 2009, 2010; Neri *et al*., 2014; Cunniffe *et al*., 2015b). Citrus canker is a disease of most species of citrus characterised by erumpent lesions on fruit, foliage and young stems. The most recent introduction of citrus canker to Florida was first detected near Miami airport in 1995 (Graham *et al*., 2004). Starting in 1998, the US government spent an estimated $1 billion on survey, control and compensation costs during a campaign that attempted to eradicate citrus canker (Gottwald *et al*., 2002a). Attempted eradication was based on an extensive programme of disease surveillance by trained teams of inspectors, aiming to detect symptomatic trees, followed by removal of all citrus canker hosts within a certain radius of detected citrus canker infection. The eradication programme resulted in the removal of at least 10 million citrus trees across Florida (Irey *et al*., 2006), from both commercial citrus groves and residential settings. However, the attempt to eradicate was controversially abandoned in May 2006 after a consensus was reached that by then the disease was too widely dispersed for eradication to continue to be a realistic proposition.

We use control of an isolated outbreak of citrus canker as a case study to address the following questions concerning epidemiologically motivated management strategies. (1) Can we develop more effective methods of control, motivated by pathogen epidemiology, that outperform constant radius removal? (2) Can the new control strategies be made sufficiently transparent to be acceptable to stakeholders? (3) How does performance depend on values of the parameters controlling disease spread, and the spatial arrangements of susceptible hosts? (4) How rapidly does performance degrade when parameters for disease spread are known only imperfectly?

## Description

### Underlying epidemiological model

**Figure 1:**
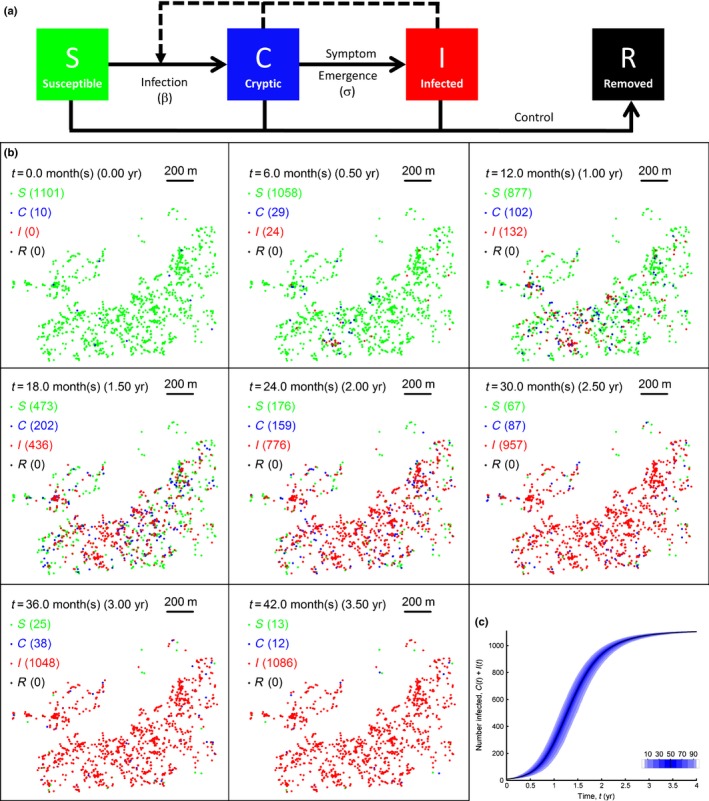
The model and its default behaviour when there is no control. (a) The underlying epidemiological model. Host plants move from susceptible (*S*) to cryptic (*C*) when first infected; from *C* to infected (*I*) as symptoms emerge; and can be removed (*R*) due to control after being detected via a survey. (b) Typical epidemic when there is no control. (c) Disease progress curve when there is no control. Shades of blue show the deciles of the distribution; black curve shows the median.

Our model tracks *N* host trees: susceptible (*S*) hosts are uninfected; cryptic (*C*) hosts are infectious but asymptomatic; infected (*I*) hosts are both infectious and symptomatic; and removed (*R*) hosts have been removed by control (Fig. 1a; Table 1). If host *i* is susceptible at time *t*, then it becomes (cryptically) infected at rate

(Eqn 1) $$\varphi_{i}\left(t\right)=\beta \sum_{j\in \left\{\Omega_{C}\left(t\right),\Omega_{I}\left(t\right)\right\}}K(d_{ij};\alpha ).$$

In this ‘force of infection’ (Keeling & Rohani, 2007), β is the rate of secondary infection and the summation index *j* runs over all infectious hosts (i.e. $\Omega_{C}\left(t\right)$ and $\Omega_{I}\left(t\right)$ represent hosts in classes *C* or *I* at time *t*, respectively). The dispersal kernel, $K(d_{ij};\alpha ),$ sets the rate of disease transmission between a pair of hosts separated by distance $d_{ij}$, and is parameterised by a scale parameter α. To allow robustness to the form of dispersal to be explored, we consider two contrasting kernels: the thin‐tailed exponential kernel, K(d;α)=exp(−d/α); and the thick‐tailed Cauchy kernel, $K\left(d;\alpha \right)=1/\left(1+{\left(d/\alpha \right)}^{2}\right)$.

**Table 1:** Definitions of symbols and default values of parameters

Symbol	Description	Default value
*t*	Time since start of the epidemic	na
*S*(*t*)	Number of susceptible (healthy) hosts at time *t*	na
*C*(*t*)	Number of cryptic (infectious but asymptomatic) hosts at time *t*	na
*I*(*t*)	Number of infected (infectious and symptomatic) hosts at time *t*	na
*R*(*t*)	Number of removed (controlled) hosts at time *t*	na
*N*	Total number of hosts	1111
$\Omega_{X}\left(t\right)$	Set of indices of hosts in compartment X ($X\in \left\{S,C,I,R\right\}$) at time *t*	na
β	Rate of secondary infection	0.00036 d^−1^
α	Scale parameter of dispersal kernel	37 m
σ	Rate of symptom emergence	1**/**107 d^−1^
*d*_*ij*_	Distance between hosts *i* and *j*	na
K(d;α)	Dispersal kernel (varies with distance *d* and scale parameter α)	Cauchy: $1/\left(1+{\left(d/\alpha \right)}^{2}\right)$
Δ	Interval between successive surveys for disease	90 d
$T_{n}$	Time of the *n* ^th^ survey	nΔ
*p*_d_	Probability of detecting symptoms	1.0
$\varphi_{i}\left(t\right)$	Force of infection on uninfected host *i* at time *t*	Eqn 1
$\tilde{\beta }$	Estimated rate of secondary infection	β
$\tilde{\alpha }$	Estimated scale parameter of dispersal kernel	α
$\tilde{\sigma }$	Estimated rate of symptom emergence	σ
$E_{i,n}$	Risk of further spread due to host *i* at time $T_{n}$	Eqn 2
$q_{i,n}$	Probability that host *i* is uninfected at time $T_{n}$	Eqn 3
$p_{i,n}^{XY}$	Estimated conditional probability of the *i* ^th^ host being in state Y by $T_{n}$ given it in state X at $T_{n-1}$	Eqn 4
${\tilde{\varphi }}_{i,n}$	Estimated force of infection on host *i* between surveys at times $T_{n-1}$ and $T_{n}$	Eqn 5
$R_{i,n}$	Estimated expected number of infections that would be caused by host *i* after time $T_{n}$, if it actually were to be infected	Eqn 6
$E_{i,n}^{\mathrm{eff}}$	Effective risk of further spread due to host *i*	Eqn 7
${\tilde{R}}_{i,0}$	Basic reproductive number (at the start of the epidemic) of host *i*, relative to the average over all hosts	Eqn 8
$R_{i}^{\max}$	Removal radius around host *i* (variable radius only)	Eqns (Eqn 9), (Eqn 10), (Eqn 11)
R*	Average removal radius (constant and variable radius only)	Optimisable threshold parameter
*E*_min_	Minimum effective risk for removal after any survey (risk‐based only)	Optimisable threshold parameter
γ	Optimisation parameter (risk‐based and variable radius only)	Optimisable threshold parameter

na, Not applicable. Parameter values are taken from Gottwald *et al*. (2001, 2002b), Cook *et al*. (2008), Parnell *et al*. (2009, 2010) and Cunniffe *et al*. (2015b).

The data used to parameterise the model were originally collected as part of a United States Department of Agriculture (USDA) study that tracked the spread of citrus canker in five populations of citrus trees in residential areas in Florida (Gottwald *et al*., 2002b; Cook *et al*., 2008; Parnell *et al*., 2009; Neri *et al*., 2014). By default we model spread and control in one of these sites, a *c*. 1 km^2^ urban region in Broward County, north of Miami, containing a host population of just over 1100 citrus trees (Gottwald *et al*., 2002b). Epidemics are initiated with 10 random individuals cryptically infected at *t *=* *0 (selecting a different set of 10 hosts on each run of the simulation), and continue until either all hosts are infected or the pathogen is eradicated. We checked via sensitivity analysis that the arbitrarily selected choice of 10 as the number of initially infected plants did not affect our qualitative results (data not shown). The pathogen disperses according to the Cauchy dispersal kernel, with scale parameter α = 37 m (Cook *et al*., 2008; Parnell *et al*., 2009, 2010). The transition from the *C* to the *I* class occurs at fixed rate σ, and represents the time taken for sufficient symptoms to emerge on a host plant for it to be detectable as symptomatic via visual inspection. We follow previous work in taking the average of this cryptic period to be 1/σ = 107 d (Cook *et al*., 2008; Parnell *et al*., 2009, 2010). For simplicity the model does not include an exposed compartment (i.e. trees that are infected but as yet not infectious or symptomatic), since the latent period of citrus canker (*c*. 7–21 d; see Gottwald *et al*., 2002b) is short relative to typical timescales for infection. The baseline infection rate β = 0.00036 d^−1^ ensures it takes *c*. 500 d for 50% of hosts to become infected (Fig. 1b; Supporting Information Video S1), reflecting typical epidemic timescales in the five USDA sites near Miami described earlier (Gottwald *et al*., 2002b). However, we test the robustness of our results to epidemiological parameters via extensive sensitivity analyses. The stochastic model is simulated using code written in the programming language C (Kernighan & Ritchie, 1988) that uses the Gillespie algorithm (Keeling & Rohani, 2007) to calculate waiting times between epidemiological transitions.

### Modelling control

Citrus canker does not kill infected trees, and so hosts only enter the removed compartment via control. We assume all hosts are surveyed regularly at intervals Δ (default ‘survey interval’ Δ = 90 d), and that symptomatic hosts are then detected with probability *p*_d_ (default ‘detection probability’ *p*_d_ = 1.0). We also assume symptomatic hosts are removed immediately after detection. The crux of what we are testing here is the performance of methods to identify additional asymptomatic hosts to remove pre‐emptively. In particular, we compare three strategies: constant control radius, risk‐based control and variable control radius.

#### Constant control radius

The constant radius strategy simply removes all hosts within a certain predetermined distance, R*, of any detected host, irrespective of perceived disease status. The rationale is that disease spread is localised, and so this targets hosts that are infected but remain asymptomatic. There is a single tuneable parameter, R*, the ‘removal radius’, which must be set in advance. This method is often used in practice (DEFRA, 2014; Peterson *et al*., 2015; Callaway, 2016; Martelli, 2016). Of particular relevance here is that this method was used for citrus canker during the joint USDA, APHIS/Florida Department of Agriculture and Consumer Services Citrus Canker Eradication Program (CCEP) in Florida between 1995 and 2005 (Gottwald *et al*., 2001).

#### Risk‐based control

The risk‐based strategy is significantly more complex. The underlying idea is to rank apparently uninfected hosts for removal based on the threat each poses to the remaining host population (te Beest *et al*., 2011). This threat could be quantified exactly if it were possible to calculate the future risk due to host *i*, $E_{i,n}$, that is, the number of secondary infections host *i* is expected to cause in the remaining host population given the information available at the time of the *n* ^th^ survey, $T_{n}=n\Delta$. The risk can be partitioned

(Eqn 2) $$E_{i,n}=q_{i,n}R_{i,n},$$

in which $q_{i,n}$ is the probability host *i* is infected at time $T_{n}$, and $R_{i,n}$ is the expected number of infections this host would cause were it infected. However, $q_{i,n}$ and $R_{i,n}$ must be estimated from the available data: the distribution of hosts, the locations of all detected hosts to date, and (potentially imprecise) estimates of the disease spread parameters $\tilde{\alpha }$, $\tilde{\beta }$ and $\tilde{\sigma }$ (where here and henceforth we use a tilde to distinguish those values of parameters used in designing control interventions from those used in simulating disease spread).

In Methods S1 we derive the following approximate formulae linking estimates of infection probabilities directly after the *n* ^th^ survey, $q_{i,n}$, to the previous set of estimates, $q_{i,n-1}$ (see also Fig. S1). We use these iteratively to update risks of infection given the information revealed by successive surveys, assuming $q_{i,0}=0$ for all *i* (i.e. all hosts are uninfected initially). The iteration is

(Eqn 3) $$\begin{array}{cc} q_{i,n}= & \frac{q_{i,n-1}p_{i,n}^{CC}+\left(1-q_{i,n-1}\right)p_{i,n}^{SC}}{q_{i,n-1}p_{i,n}^{CC}+\left(1-q_{i,n-1}\right)\left(p_{i,n}^{SC}+p_{i,n}^{SS}\right)},\;\;\text{for}\;\;\;1\leq i\leq N,n\geq 1, \end{array}$$

in which

(Eqn 4) $$\begin{array}{cc} p_{i,n}^{CC} & =\exp(-\tilde{\sigma }\Delta ),\\ p_{i,n}^{SS} & =\exp(-{\tilde{\varphi }}_{i,n}\Delta ),\\ p_{i,n}^{SC} & =\frac{{\tilde{\varphi }}_{i,n}}{{\tilde{\varphi }}_{i,n}-\tilde{\sigma }}\left(\exp\left(-\tilde{\sigma }\Delta \right)-\exp\left(-{\tilde{\varphi }}_{i,n}\Delta \right)\right), \end{array}$$

and where the estimated force of infection on host *i* between surveys at times $T_{n-1}$ and $T_{n}$ is

(Eqn 5) $${\tilde{\varphi }}_{i,n}=\tilde{\beta }\sum_{j\in \left\{I(T_{n})\right\}}K(d_{ij};\tilde{\alpha }).$$

Our estimate of the future number of potential infections that would be caused by host *i* were it to be infected is (cf. Methods S1)

(Eqn 6) Ri,n=Δβ~∑j∈S(Tn),C(Tn)j≠iK(dij;α~).

In Eqns 5 and 6, $S(T_{n})$, $C(T_{n})$ and $I(T_{n})$ are the sets of host plants that are susceptible, cryptically infected and symptomatically infected, respectively, all at time *T*_*n*_. We note that in driving the management strategy, the true disease status of cryptically infected hosts is incorrectly accounted for (because effectively these hosts are erroneously assumed to still be susceptible). We also note that when detection is imperfect (*p*_d_ < 1.0), then Eqns 5 and 6 are updated in the obvious fashion, that is, to use only detected hosts to set the estimated force of infection, but to calculate the number of potential infections by considering all hosts that are not already removed or just detected as infected.

The approximations involved in estimating $q_{i,n}$ and $R_{i,n}$ are based only on the data revealed by successive rounds of surveillance, and so are imperfect, which indicates the estimated risk $E_{i,n}$ is not exact. Exploratory work indicated performance of risk‐based control could be improved by ranking hosts in terms of values of a related quantity, the ‘effective risk’

(Eqn 7) $$E_{i,n}^{\mathrm{eff}}=q_{i,n}{\left[R_{i,n}\right]}^{\gamma },$$

in which $R_{i,n}$ is raised to the power γ. This additional ‘bias’ parameter controls the relative importance of the probability of infection vs the likelihood of further spread in setting the risk posed by a host. Large values of γ correspond to prioritising hosts with large capacity for onwards spread, irrespective of (estimated) disease status. Small values of γ prioritise hosts that are estimated to be more likely to be infected, irrespective of whether they would then be expected to cause many future infections.

Given estimates of the effective risk, the risk‐based strategy uses the following algorithm to distribute culls.

1. Remove all detected hosts.
2. Find the host j* with the largest effective risk, E*.
3. While E* > *E*_min_, repeat the following.
  - Remove host j*.
  - Recalculate $R_{j,n}$ and so $E_{j,n}^{\mathrm{eff}}$ for each remaining host, *j*.
  - Find the host j* with the largest effective risk, E*.

Step 3(b) is required because the future risks of infection are affected by host removal. Performance of the risk‐based strategy depends on two tuneable parameters, *E*_min_ and γ.

#### Variable control radius

The risk‐based strategy depends in a complex fashion on the pattern of spread and removals to date, and this might not be transparent for some stakeholders. The variable radius strategy provides a simpler approximate method to determine hosts to remove, which would potentially be easier for stakeholders to implement and/or understand. The strategy seeks to translate local variations in host density at the start of the epidemic into fixed estimates of the threat posed by each host should it become infected. These are used to set a host‐specific removal radius, $R_{i}^{\max}$ *,* within which hosts are removed around the *i* ^th^ host if it is indeed detected. However, importantly for implementation and potentially for acceptability and uptake by stakeholders, host‐specific removal radii do not change as the epidemic evolves, and so could be distributed in advance of any epidemic.

In Methods S2 we derive an expression for $R_{i}^{\max}$ that depends on ${\tilde{R}}_{i,0}$, the host's basic reproductive number at the start of the epidemic, relative to the average over all hosts

(Eqn 8) $${\tilde{R}}_{i,0}=\frac{\sum_{j\neq i}K(d_{ij};\tilde{\alpha })}{\frac{1}{N}\sum_{k}\left(\sum_{j\neq k}K(d_{jk};\tilde{\alpha })\right)}.$$

For the Cauchy dispersal kernel, after defining

(Eqn 9) $$\theta_{i}={\left[{\tilde{R}}_{i,0}\right]}^{\gamma }\left(1+{\left(\frac{R^{*}}{\tilde{\alpha }}\right)}^{2}\right),$$

the control radius for host *i* is then (cf. Methods S2)

(Eqn 10) $$R_{i}^{\max}=\left\{\begin{array}{cc} \tilde{\alpha }\sqrt{\theta_{i}-1} & \text{if}\;\theta_{i}>1\\ 0 & \text{otherwise} \end{array}\right..$$

For the exponential dispersal kernel

(Eqn 11) $$R_{i}^{\max}=\max\left(R^{*}+\gamma \tilde{\alpha }\log\left({\tilde{R}}_{i,0}\right)\;,0\right).$$

In both cases the radii depend on configurable parameters R* and γ, and $R_{i}^{\max}$ = 0 corresponds to simply roguing the host in question.

### Epidemic size

If control is not attempted, all hosts will eventually become infected (becasuse the rapid spread of citrus canker allows us to assume there is no ‘natural’ death of hosts in the absence of disease in our underlying model). However, when control is attempted it is likely disease will be eradicated before all hosts have become infected. We then define the (final) ‘epidemic size’ to be the number of removed trees at the time of eradication, at which time all hosts either remain healthy or are removed. We use the epidemic size as a convenient metric to compare the performance of the different control strategies considered here.

### Host landscapes and dispersal kernels

We test the robustness of our methodology to the form of the dispersal kernel by examining performance when epidemics spread via an exponential (rather than Cauchy) dispersal kernel. In addition to the default landscape directly mapped from the USDA Miami experimental sites, we also test the strategies on two alternative host landscapes: a Random landscape, in which 2000 hosts are scattered uniformly across a 1 km^2^ area; and an Orchard landscape, which has 2016 hosts in two adjacent blocks, planted in rows 10 m apart and with a 5 m within‐row host spacing (a spacing consistent with practice in the US citrus industry in Florida).

To isolate the effects of changes caused by the dispersal kernel or host layout, we fix the values of the epidemiological and detection parameters to be as for the default landscape and kernel. However, in the model we use here, values of the infection rate parameter, β, must be normalised to transfer between landscape–kernel combinations (Cunniffe *et al*., 2015b). We therefore select baseline values of β that ensure the average time for infection of half the hosts in the landscape is 500 d. The procedure led to the following rates: Miami B2‐Exponential, β = 0.0009 d^−1^; Random‐Cauchy, β = 0.00028 d^−1^; Orchard‐Cauchy, β = 0.000057 d^−1^ (cf. β = 0.00036 d^−1^ for Miami B2‐Cauchy). However, we note we test robustness to changes in the rate of secondary infection, β (and the scale of the dispersal kernel, α), for all three additional landscape–kernel combinations we consider. In all cases the dispersal parameter α ranges from 10 to 70 m; the ranges of values used for rate of secondary infection β depend on the landscape–kernel combination under consideration, but in all cases corresponds to values from 10 to 250% of the baseline infection rate.

## Results

### Baseline performance

**Figure 2:**
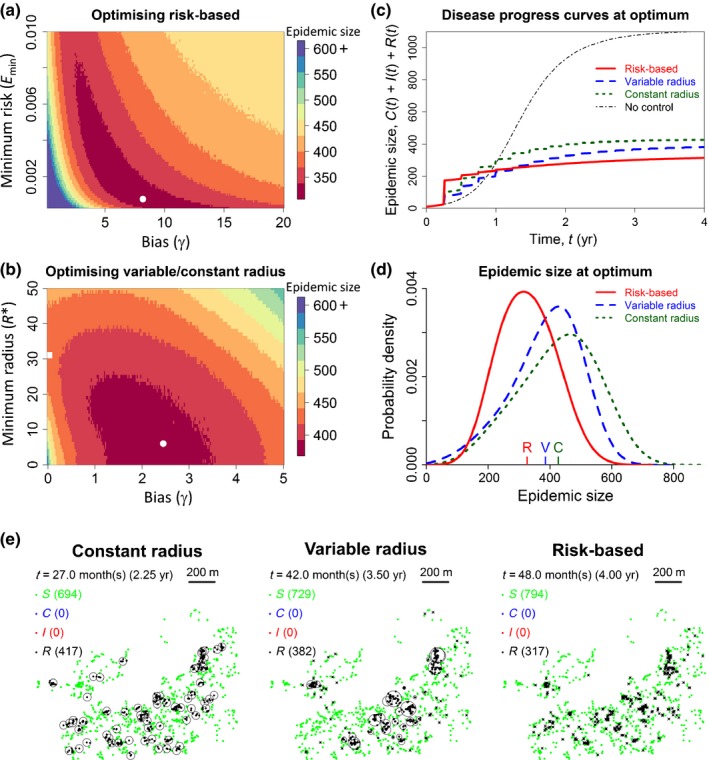
Risk‐based control outperforms variable radius control, which outperforms constant radius control. (a) Optimising the risk‐based strategy; the optimised threshold and bias parameters, which lead to the smallest average epidemic size (i.e. number of hosts removed by the end of the epidemic when the pathogen is eradicated), are *E*_min_ = 0.00075 and γ = 8.2, respectively (marked with a white circle). (b) Optimising the variable radius strategy: the optimal values R* = 6 m and γ = 2.45 are marked with a white circle. The optimal constant radius strategy can be identified from this plot by considering only values of R* with γ = 0 (cf. Eqns 10, 11); this value, R* = 31 m, is marked with a white square. (c) Disease progress curves at the optima identified in (a) and (b), showing the mean of 5000 simulation runs for each strategy for each time. (d) Probability distributions of the final epidemic size for each strategy using the optimised parameters, showing the variability in the eventual total number of removals. The mean epidemic sizes are marked by the letters just above the *x*‐axis. (e) State at the end of a randomly chosen epidemic with control for each control strategy. The black circles show the removal radii around particular hosts; crosses denote a removal radius of zero. Full time‐courses of these particular (indicative) epidemics are given in Supporting Information Videos [Link], [Link], [Link].

Control strategies were compared by optimising the bias (γ) and threshold (R* or *E*_min_) parameters via exhaustive searches, using mean epidemic size to compare performance (Fig. 2a,b; Videos S2–4). The risk‐based strategy (*E*_min_ = 0.00075, γ = 8.2) led to an average of 326.1 host removals by the time of eradication (Table 2), a 23.7% improvement over the optimal constant radius strategy (R* = 31 m; mean epidemic size = 427.6). Improvement under the variable radius strategy was smaller, but nevertheless there was a 9.8% decrease in epidemic size relative to constant radius control (R* = 6 m, γ = 2.45; mean epidemic size = 385.9). For both the variable radius and (particularly) the risk‐based strategy, different ranges of values of the parameters R* and *E*_min_ become relevant as potential thresholds for different values of the bias parameter, γ, leading to complex contours of constant epidemic size (Fig. 2a,b). However, as each strategy was optimised via an exhaustive search over a large range of possible values of the threshold and bias parameters, correct optima could nevertheless be identified reliably.

**Table 2:** Summary of the performance of the three control strategies on all four landscape–kernel combinations

		Miami B2 (Cauchy)	Miami B2 (Exponential)	Random (Cauchy)	Orchard (Cauchy)
Constant radius	Optimum R*	31 m	36 m	64 m	16 m
	Mean epidemic size (2.5%, 50%, 97.5%)	427.6 (178, 439, 638)	324.3 (139, 322, 521)	1200.5 (654, 1227, 1589)	1464.5 (1136, 1487, 1681)
Variable radius	Optimum R*	6 m	30 m	42 m	2 m
	Optimum γ	2.45	1.40	4.75	2.85
	Mean epidemic size (2.5%, 50%, 97.5%)	385.9 (143, 403, 549)	310.4 (133, 310, 500)	1088.7 (587, 1128, 1385)	1152.0 (802, 1181, 1337)
	Mean improvement	9.8%	4.3%	9.3%	21.3%
Risk‐based	Optimum *E*_min_	0.00075	0.016	0.00025	0.00025
	Optimum γ	8.2	1.8	8.9	8.1
	Mean epidemic size (2.5%, 50%, 97.5%)	326.1 (192, 323, 486)	279.2 (130, 278, 440)	881.7 (614, 895, 1111)	949.0 (732, 953, 1162)
	Mean improvement	23.7%	13.9%	26.6%	35.2%

Selected percentiles (2.5%, 50%, 97.5%) of the full distribution of the number of hosts removed at optimum performance are given. Mean improvement refers to the percentage difference in means between the risk‐based or variable radius strategies and the constant radius strategy, as a percentage of the mean epidemic size under the constant radius strategy.

For both of the epidemiologically motivated strategies, optimal bias parameters γ > 1 suggest optimisation emphasises pre‐emptive removal of hosts predicted to cause many infections (cf. Eqn 7). This is confirmed by the (average) disease progress curves at optimum performance (Fig. 2c) and animations of the model (Video S4). Although the risk‐based strategy eventually results in many fewer removals, an average of just under 175 hosts are pre‐emptively removed on the first survey (16% of all hosts in the landscape; just over 50% of all removals). Removals under the other strategies only surpasses this after some time. Distributions of final epidemic sizes corresponding to optima for each strategy reveal wide variability in final sizes, reflecting the inherent variability of pathogen spread and control as represented in our stochastic model (Fig. 2d). However, while distributions of epidemic sizes overlap, differences in the effectiveness of the strategies are evident.

### Robustness to epidemiological and logistical parameters

**Figure 3:**
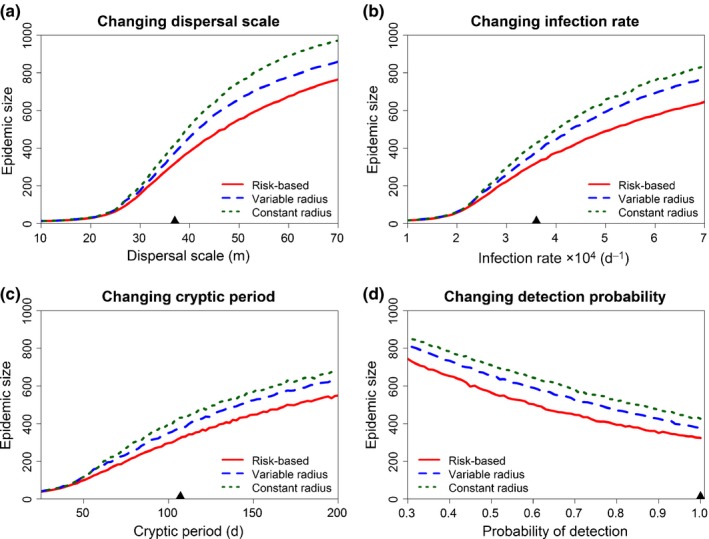
The relative performance of the control strategies does not depend on values of epidemiological and management parameters (when they are known in advance of the epidemic). (a) Response of the optimal performance of the three control strategies to the dispersal scale, independently optimising the performance of each strategy at each value of the dispersal parameter (i.e. repeating the process underlying Fig. 2(a,b) for each dispersal scale, α). The mean epidemic size (i.e. mean number of hosts removed by the time of eradication) at optimum is shown on the *y*‐axis of the graph, and the default dispersal scale is marked by the black triangle on the *x*‐axis. (b) As for (a), but showing response to the rate of secondary infection, β. (c) As for (a), but showing response to the average cryptic period, 1/σ. (d) As for (a), but showing response to the probability of detecting symptomatic hosts in a single round of surveying, *p*_d_.

We tested the effect of varying four epidemiological parameters around their default values (cf. Table 1): dispersal scale (α; default value, 37 m), infection rate (β; default value, 0.00036 d^−1^), average cryptic period (1/σ; default value, 107 d) and probability of detection ($p_{d}$; default value 0.8) (Fig. 3). For each value of one of these parameters, with the other three parameters fixed constant at the default value, optimal values of the threshold and bias parameters were found, again by exhaustive search to minimise mean epidemic size. The relative ordering of the strategies was unchanged, with the risk‐based strategy consistently outperforming the variable radius strategy, which in turn outperformed the constant radius strategy. This pattern was unchanged over all values of all parameters we considered, apart from when epidemics spread very slowly and/or were otherwise very easy to control due to short cryptic periods, in which case the performances of the different strategies were indistinguishable (cf. small values of the independent variable in Fig. 3a–c). The relative performance of the different strategies is thus extremely robust to parameter changes, at least when parameter values are known precisely.

### Robustness to misspecification of parameters

The results presented in Fig. 3 correspond to a situation in which there is perfect knowledge of epidemiological parameters. Of more practical interest is the robustness of the different control strategies when epidemiological parameters are known only imprecisely. This introduces two sources of error: the parameters used in the simulations to optimise threshold and bias parameters are imperfectly known; and the parameters driving control before and/or during the epidemic (cf. Eqns 7 and 10 or 11) would also be imperfect.

**Figure 4:**
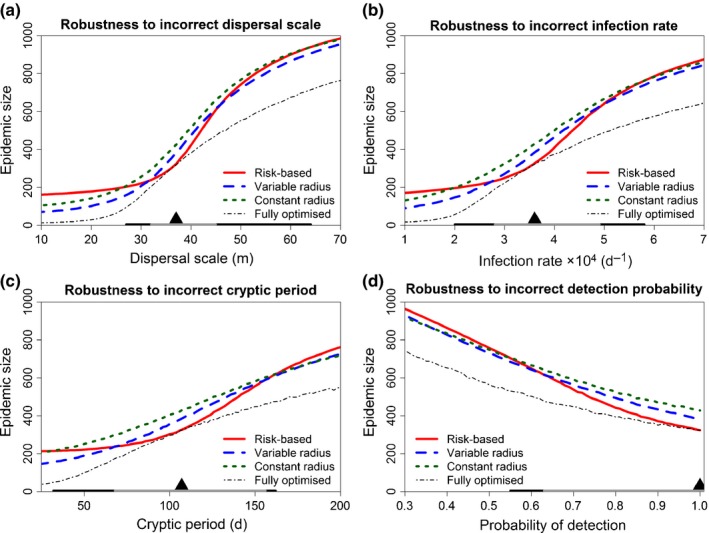
Performance of the risk‐based and variable radius control strategies degrades if parameters are not known in advance of the epidemic. (a) Response of the performance of the three control strategies to changes in the dispersal scale, when the control strategies were optimised incorrectly using the default dispersal scale, and when this default scale is used during the epidemic to calculate $E_{i,n}^{\mathrm{eff}}$ (Eqn 7 and $R_{i}^{\max}$ (Eqn 10). The average epidemic size (i.e. mean number of hosts removed by the time of eradication) for the risk‐based strategy when optimised correctly is shown for comparison (dash‐dotted line). The mean epidemic size is shown on the *y*‐axis of the graph, and the default dispersal scale is marked by the black triangle on the *x*‐axis. The range of dispersal scales for which the (incorrectly optimised) risk‐based strategy outperforms the (incorrectly optimised) variable radius strategy is marked by the grey shading along the *x‐*axis. The range for which the risk‐based strategy outperforms the (incorrectly optimised) constant radius strategy but is outperformed by the (incorrectly optimised) variable radius strategy is shown by the black shading. (b) As for (a), but for misspecification of the rate of secondary infection. (c) As for (a), but for misspecification of the average cryptic period. (d) As for (a), but for misspecification of the probability of detecting symptomatic hosts.

We tested the effect of parameter misspecification by allowing the epidemiological parameters controlling the epidemic (i.e. α, β, 1/σ and $p_{d}$) to vary, while fixing these parameters at their default values (α = 37 m, β = 0.00036 d^−1^, 1/σ = 107 d and $p_{d}$ = 0.8) to drive the control strategies, and using the optimal values of the threshold and bias parameters derived from the default parameter set (i.e. the values R* = 31 m (constant radius) or 6 m (variable radius), *E*_min_ = 0.00075 (risk‐based) and γ = 2.45 (variable radius) or 8.2 (risk‐based) highlighted in Fig. 2a,b). For all four parameters we tested, the relative performance of both epidemiologically motivated strategies degrades as the degree of misspecification increases (Fig. 4). As estimates of parameters become progressively more imprecise, the risk‐based control becomes less effective than the variable radius strategy. If the lack of knowledge is sufficiently severe, then the risk‐based control is even outperformed by the simple constant radius strategy. The risk‐based strategy is most dependent on precise estimates of epidemiological parameters, which is unsurprising, since the reason it outperforms the other strategies is that it includes the most epidemiological information.

### Robustness to host landscape and dispersal kernel

**Figure 5:**
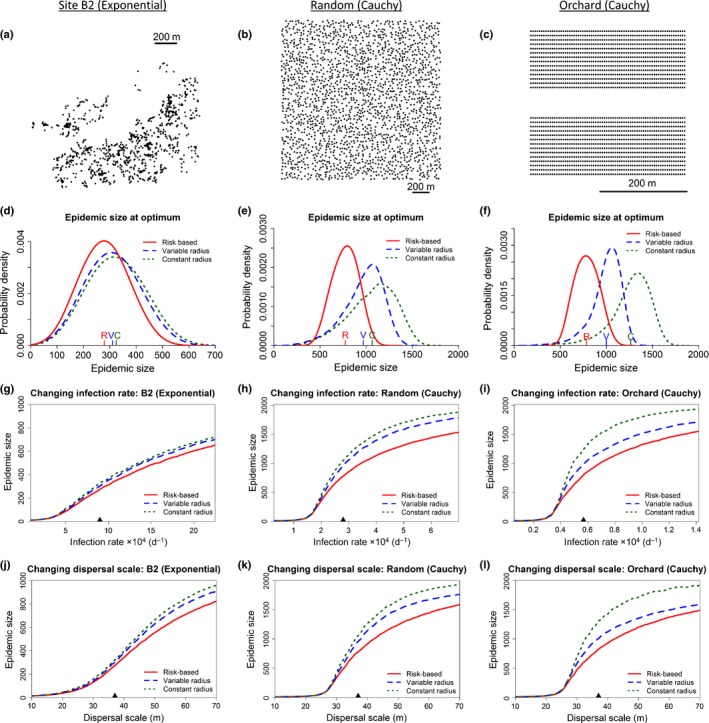
The relative performance of the control strategies does not depend on host landscape structure, but the improvement from risk‐based control is smaller when dispersal is thin‐tailed. (a–c) Host landscapes and dispersal kernel combinations used to assess the robustness of the methods: Miami Broward County Site B2 using an exponential dispersal kernel; a random landscape consisting of 2000 hosts randomly positioned over 1 km^2^ (with Cauchy dispersal); and a small citrus orchard, consisting of 2016 hosts at a regular spacing (with Cauchy dispersal). (d–f) Full probability distributions of the epidemic sizes at optimum. Mean epidemic size (i.e. mean number of hosts removed by the time of eradication) for each strategy is marked by letters just above the *x*‐axis of each plot. (g–i) Responses of average epidemic size at optimum to changes in the rate of secondary infection. The default rate of secondary infection is marked with a triangle on the *x*‐axis of each plot. (j–l) Responses of average epidemic size at optimum to changes in the scale parameter of the dispersal kernel. Again the default value is marked with a triangle.

For all three additional landscape–kernel combinations we tested, the relative performance of the three strategies is unchanged, with risk‐based control outperforming the variable radius strategy, which in turn outperforms the constant control radius strategy (Fig. 5d–f; Table 2). The behaviour as the scale of dispersal and the infection rate are increased is also similar; because larger epidemics are harder to control, the additional efficiency of the risk‐based control strategy leads to larger improvements (Fig. 5g–l). Although the risk‐based strategy did consistently outperform the other strategies when dispersal was characterised by the thin‐tailed exponential kernel (Fig. 5d,g,j), differences between strategies were then less stark.

## Discussion

The well‐documented rise in the number of emerging diseases of plants, combined with the impact of and difficulty associated with eradication, underpins the urgent need for more efficient interventions. The idea underlying this paper is that reactive control of plant disease can be made more efficient by including additional epidemiology, thereby going beyond simply treating all hosts within a certain distance of detected infection. Our key result is that removal of hosts judged to pose a high risk of transmitting disease in the future can significantly outperform constant radius removal in terms of reducing epidemic size. The risk of future transmission is estimated as the epidemic progresses by combining estimates of the probability that an asymptomatic host is infected with estimates of the number of remaining hosts it would then infect. Prioritising hosts for removal based on the risk posed to the rest of the population is robust to changes in the precise values of parameters controlling disease spread and to the pathogen's dispersal kernel. The strategy is also robust to patterns of susceptible hosts in the landscape, although it does require good advance knowledge of epidemiological parameters to be successful. By approximating the risk of future infection by static estimates of the threat posed by each host should it become infected, the risk‐based strategy can also be translated into a simpler variable radius strategy. The variable radius strategy has the advantage that the set of hosts to be removed does not depend in such a complex fashion on patterns of detected hosts, and so the strategy is potentially more acceptable to affected stakeholders. We have shown that this variable control radius strategy has intermediate performance between risk‐based and constant radius control, again for a wide range of epidemiological parameters and host landscapes.

We framed our analysis in terms of controlling a localised outbreak of citrus canker, caused by the bacterium *X. citri*. This is an evocative case study: the US government spent over $1 billion between 1995 and 2005 in an (ultimately unsuccessful) attempt to eradicate citrus canker from Florida (Gottwald & Irey, 2007), removing over 10 million citrus trees from homeowners’ gardens and commercial orchards in the process (Gottwald *et al*., 2002a). Management was based on removal of all citrus canker hosts within a fixed radius of detected infection, equivalent to the constant radius strategy considered here. Focusing on citrus canker allowed us to use a detailed pre‐existing data set on disease spread in the Miami region, originally collected by the USDA (Gottwald *et al*., 2002b). A model of the spread of citrus canker has been successfully fitted to these data, and we used that model here, thereby following several previous studies that have considered constant radius management strategies (Cook *et al*., 2008; Parnell *et al*., 2009, 2010; Cunniffe *et al*., 2015b). While the underlying model has been extensively validated for the spread of citrus canker in Miami (Neri *et al*., 2014), it is flexible, and has been used for several other plant diseases, including huanglongbing (also known as citrus greening) (Parry *et al*., 2014; Cunniffe *et al*., 2015b; Parnell *et al*., 2015), Bahia bark scaling (Cunniffe *et al*., 2014) and sudden oak death (Demon *et al*., 2011; Thompson *et al*., 2016a).

We compared control strategies in terms of the total number of hosts removed before total eradication of disease. Other metrics could be appropriate, including the duration of the epidemic, the potential for export of inoculum to distant populations, or the combined cost of detection and control. The duration of the epidemic is related to the tolerance of stakeholders for control: the longer an epidemic continues within a commercial or residential landscape, the more fragile the resolve of the impacted stakeholders affected by the eradication programme. It also has consequences for trade if quarantine measures are put in place until eradication can be demonstrated. We note that for the metrics related to epidemic duration, both the risk‐based and the variable radius strategies are outperformed by simple constant radius control, at least when all three strategies are optimised to minimise the number of removals (mean epidemic durations: constant radius = 2.48 yr, variable radius = 3.89 yr, risk‐based = 4.55 yr). However, it is clearly impossible to develop management strategies that optimise all possible metrics simultaneously (Probert *et al*., 2016). Taking a simple example, epidemic duration would certainly be minimised by immediate removal of all hosts, but this clearly would not scale to successful management at larger scales. We therefore focused here on what we consider to be the most pressing consequence of a control strategy: the number of hosts lost before the epidemic is controlled. Nevertheless, an interesting extension to the work presented here – and one echoing the challenge faced by policy‐makers in practice – would be to develop techniques to select management strategies by optimising a weighted combination of different metrics (Cunniffe *et al*., 2015b).

Improved performance of the risk‐based and variable control radius strategies is robust to changes to epidemiological parameters (Fig. 3) and to the pattern of host plants through which the epidemic spreads, at least when using the (fat‐tailed) Cauchy dispersal kernel (Fig. 5). However, when dispersal follows an exponential kernel, relatively large dispersal scales are required for risk‐based control to attain a high level of improvement relative to constant radius control. Exponential dispersal leads to wave‐like spread of disease, with well‐defined spreading foci of infection (Shaw, 1995). The use of a constant radius for control is then difficult to improve upon, because disease spread is relatively predictable and localised, and so distance is a very good proxy for risk of infection. This interpretation is supported by the improved relative performance of the risk‐based strategy when the dispersal scale of the exponential kernel becomes longer, because local disease spread is then less tightly restricted to neighbourhoods of existing foci, and so the set of plausible subsequent infections from any infected host becomes more widely spaced (Fig. 5j). We note that fat‐tailed kernels are more often supported by the patchy patterns of spread in disease data (Cook *et al*., 2008; Meentemeyer *et al*., 2011; Filipe *et al*., 2012; Neri *et al*., 2014), and so in practice better relative performance for fat‐tailed kernels might not be a significant limitation. Epidemics characterised by fat‐tailed dispersal kernels are also acknowledged to be more difficult to control using the constant radius strategy than those that spread via thin‐tailed kernels (Cunniffe *et al*., 2015b), and so are the case for which more advanced methods are most sorely required. In passing we note there appear to be systematic differences between landscapes when there was Cauchy dispersal, such that the epidemiologically motivated strategies are more or less successful for certain landscapes (compare the degree of overlap between distributions in Figs 2d, 5e,f). We did not explore this further, because the methods developed here allow performance to be tested in advance, and so could be adopted (or not) depending on whether they are expected to be successful in the particular case of interest.

The control strategies tested here are based on the idea of ranking hosts according to the risk of infection, which we defined to be the expected number of hosts a host would be expected to cause to become infected in the future. Of course, this is not the only way of introducing additional epidemiological insight into control strategies. Work on foot‐and‐mouth disease has often concentrated on ‘predictive vaccination’, attempting to identify farms at risk of infection after two generations of spread from a central focus for pre‐emptive treatment (Keeling *et al*., 2003; Tildesley *et al*., 2006). Other work for foot‐and‐mouth disease has used a similar notion of risk to that used here to identify farms to be treated (te Beest *et al*., 2011), although with simpler estimates of the probability of infection, no allowance for biasing the probability of infection vs the number of likely future transmissions (i.e. the bias parameter, γ in our work) and not considering how the strategies could be simplified for practical use (i.e. our variable radius strategy). Other methods could also of course be tested. For example, a simple strategy which would be expected to outperform constant radius culling on the orchard landscape would be culling in ellipses rather than circles, with preferential removal of hosts within rows, since disease would be expected to spread more quickly in this direction. Another possibility would be to combine strategies, for example using a single round of largely pre‐emptive removal (i.e. ‘thinning’) of hosts that pose high risk at the time of first detection followed by constant or variable radius removal thereafter. Our purpose here, however, was to show how epidemiologically motivated strategies could be successful, rather than attempting an exhaustive characterisation of ways in which improved control could potentially be achieved.

Ever‐increasing rates of introduction of new and emerging plant diseases indicate more efficient control measures are urgently required. We have shown how including epidemiological intelligence in management strategies can reduce impacts on host populations. However, the more sophisticated methods rely upon accurate characterisation of pathogen dynamics (cf. Fig. 4) and plant host populations at risk (cf. Fig. 5), and for the methods as presented here, this must be done in advance. Nevertheless, new methods to detect and map plant diseases and host populations, as well as to characterise spread, including recent significant advances in disease diagnostics (Fang & Ramasamy, 2015), remote sensing (Martinelli *et al*., 2015) and parameter estimation (Parry *et al*., 2014), all show considerable promise. In particular, developments in diagnostics and remote sensing suggest that cryptic infections will potentially become apparent more rapidly, allowing more sophisticated disease management to become routine. These and other developments indicate the types of methods presented here will become increasingly possible in the coming years, particularly in the light of the increasing adoption and acceptance of mathematical models by policy‐makers (DEFRA, 2014). However, as we have alluded to via our variable control radius strategy, careful attention will need to be devoted to stakeholder acceptability. Additionally, real‐time adaptive management approaches to control (Shea *et al*., 2014), in which information concerning pathogen dynamics collected during a control programme is used to refine it, will become increasingly possible. In part this will be because of the increased speed with which diagnostic results can be determined in the field, including via citizen science (Meentemeyer *et al*., 2015). Allowing for adaptive management and scaling the methods up to regional‐scale management programmes, as well as continuing to examine simpler ‘rules‐of‐thumb’ to approximate complex epidemiologically motivated control strategies, will be the focus of our future work.

## Author contributions

N.J.C., R.J.O.H.S. and S.R.H‐T. planned and designed the research, with input from S.P., T.R.G. and C.A.G. S.R.H‐T. and N.J.C. performed the experiments and analysed the data. N.J.C. wrote the manuscript, with input from all co‐authors.

## Supporting information

Please note: Wiley Blackwell are not responsible for the content or functionality of any Supporting Information supplied by the authors. Any queries (other than missing material) should be directed to the *New Phytologist* Central Office.

**Fig. S1** Possible transitions that an individual host can make between successive surveys.
**Methods S1** Derivation of the risk‐based control strategy.
**Methods S2** Derivation of the variable radius control strategy.

**Video S1** A single realisation of epidemic spread with no control on the default landscape (Miami B2) and with the default epidemic spread parameters (cf. Fig. 1b).

**Video S2** A single realisation of constant radius control on the default landscape (Miami B2) and with the default epidemic spread parameters, using optimum control parameter R* = 31 m (cf. Fig. 2e).

**Video S3** A single realisation of variable radius control on the default landscape (Miami B2) and with the default epidemic spread parameters, using optimum control parameters R* = 6 m and γ = 2.45 (cf. Fig. 2e).

**Video S4** A single realisation of risk‐based control on the default landscape (Miami B2) and with the default epidemic spread parameters, using optimum control parameter *E*_min_ = 0.00075 and γ = 8.2 (cf. Fig. 2e).
