# Supplementary material for: Risk‐based management of invading plant disease
Source: New Phytol. 2017 Mar 28;214(3):1317–29. doi: 10.1111/nph.14488 (PMC5413851; doi:10.1111/nph.14488)
Supplement: Supplementary file 1 — Fig. S1 Possible transitions that an individual host can make between successive surveys. Methods S1 Derivation of the risk‐based control strategy. Methods S2 Derivation of the variable radius control strategy. [file NPH-214-1317-s005.pdf]

New Phytologist

New Phytologist Supporting Information Fig. S1 and Methods S1 & S2

Article title: Risk-based management of invading plant disease

Authors: Samuel R. Hyatt-Twynam, Stephen Parnell, Richard O. J. H. Stutt, Tim R. Gottwald, Christopher A. Gilligan and Nik J. Cunniffe

Article acceptance date: 19 January 2017

The following Supporting Information is available for this article:

Fig. S1 Possible transitions that an individual host can make between successive surveys.

Methods S1 Derivation of the risk-based control strategy.

Methods S2 Derivation of the variable radius control strategy.

Videos S1 A single realisation of epidemic spread with no control on the default landscape (Miami B2) and with the default epidemic spread parameters (cf Fig. 1b).

Videos S2 A single realisation of constant radius control on the default landscape (Miami B2) and with the default epidemic spread parameters, using optimum control parameter R^* = 31 m (cf Fig. 2e).

Videos S3 A single realisation of variable radius control on the default landscape (Miami B2) and with the default epidemic spread parameters, using optimum control parameters R^* = 6 m and γ = 2.45 (cf Fig. 2e).

Videos S4 A single realisation of risk-based control on the default landscape (Miami B2) and with the default epidemic spread parameters, using optimum control parameter E_{min} = 0.00075 and γ = 8.2 (cf Fig. 2e).

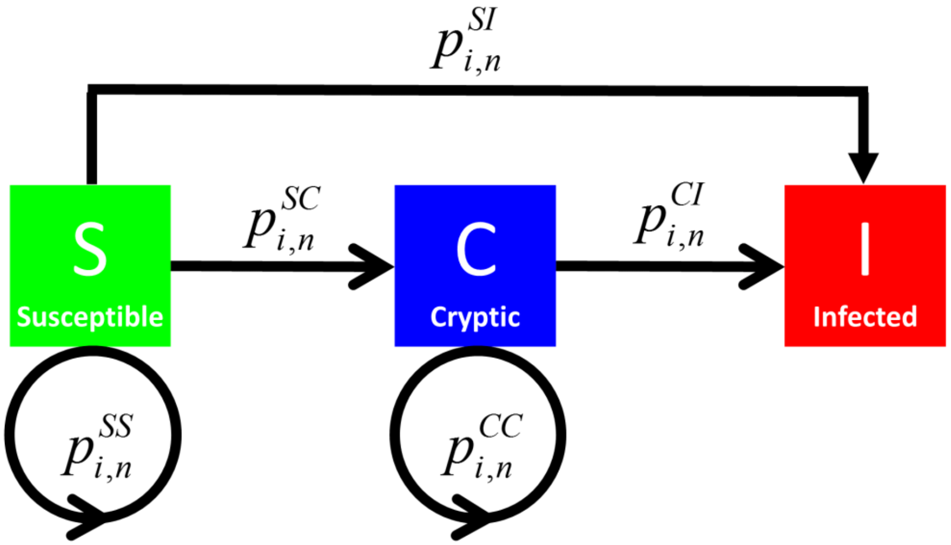

Fig. S1 Possible transitions that an individual host can make between successive surveys. The conditional probabilities $p_{i,n}^{XY}$ correspond to the probability (for the ith host) of being in state Y by the time of the nth survey given that it was in state X at the time of the $(n\text{-}1)^{\text{th}}$ survey. Note that the direct S to I transition requires the host to be in state C for some period during the survey interval.

Methods S1 Derivation of the risk-based control strategy.

Estimating probabilities of infection

The risk-based control strategy relies on estimating $q_{i,n}$, the probability that seemingly healthy host *i* is in fact infected at the time of the *n*th survey, $T_n = n\Delta$. Assuming – to derive the method – detection is perfect, and so that the probability of detecting a symptomatic host p_d = 1, then any host that is not detected at time T_n must be either susceptible or cryptic. Therefore for $1 \leq i \leq N$ and $n \geq 1$

$$\begin{aligned} q_{i,n} &= \frac{\Pr(C)}{\Pr(S)+\Pr(C)}, \\ &= \frac{q_{i,n-1}p_{i,n}^{CC} + (1-q_{i,n-1})\,p_{i,n}^{SC}}{(1-q_{i,n-1})\,p_{i,n}^{SS} + \left(q_{i,n-1}p_{i,n}^{CC} + (1-q_{i,n-1})\,p_{i,n}^{SC}\right)}, \\ &= \frac{q_{i,n-1}p_{i,n}^{CC} + (1-q_{i,n-1})\,p_{i,n}^{SC}}{q_{i,n-1}p_{i,n}^{CC} + (1-q_{i,n-1})(p_{i,n}^{SC} + p_{i,n}^{SS})}, \end{aligned} \qquad \text{Eqn S1}$$

in which $p_{i,n}^{XY}$ corresponds to the conditional probability of the *i*th host being in state Y by the time of the *n*th survey given it was in state X at the time of the (*n*-1)th survey (Fig. S1). If the quantities $p_{i,n}^{XY}$ can be estimated from spread data and estimated epidemiological parameters, Eqn S1 provides an iterative method to update the values of $q_{i,n}$ (assuming that $q_{i,0}$ = 0 for all *i*, corresponding to assuming all hosts are healthy at the start of the epidemic).

The probability that a host remains cryptic ($p_{i,n}^{CC}$) requires a cryptically infected host to not become symptomatic between rounds of surveillance. Since transitions between states are exponentially distributed,

$$p_{i,n}^{CC} = \exp(-\tilde{\sigma}\Delta), \qquad \text{Eqn S2}$$

in which $\tilde{\sigma}$ is the estimate of the rate at which symptoms emerge, and Δ is the interval between surveys. Note that this probability is independent of the values of *i* and *n*.

The transition from state S to state C is more complex. The force of infection on host *i* at time *t*, $\varphi_i(t)$ (see Eqn 1) is highly time-dependent, varying with the number and position of infected hosts. In estimating the probabilities of infection, we instead imagine the force of infection on host *i* between the survey times T_{n-1} and T_n is constant, and is controlled only by the hosts that were detected at $t = T_n$. Our estimate of the force of infection is then

$$\varphi_{i,n} = \tilde{\beta} \sum_{j \in \{I(T_n)\}} K(d_{ij}; \tilde{\alpha}), \qquad \text{Eqn S3}$$

in which $\tilde{\alpha}$ and $\tilde{\beta}$ are the estimated values of the epidemiological parameters. Assuming further that this force of infection acts for the entire interval between surveys, the probability of host *i* escaping infection is

$$p_{i,n}^{SS} = \exp(-\varphi_{i,n}\Delta). \qquad \text{Eqn S4}$$

This approximation makes two opposing simplifications. It is an over-approximation in as much as it assumes all detected hosts have been infected for the entire survey interval, but it is an under-approximation in as much as it does not account for the effect of cryptically infected hosts.

The final quantity required in Eqn S1 is $p_{i,n}^{SC}$ the probability a host starting in state S transitions to state C at some point in the interval between successive surveys, but then remains cryptic thereafter. This can be calculated (for host *i*) by convolution over all possible times during the interval, τ, at which the transition from S to C could occur

$$p_{i,n}^{SC} = \int_{\tau=0}^{\Delta} \varphi_{i,n} \exp(-\varphi_{i,n}\tau)\exp(-\tilde{\sigma}(\Delta-\tau))d\tau,$$
$$= \frac{\varphi_{i,n}}{\varphi_{i,n}-\tilde{\sigma}}\left(\exp(-\tilde{\sigma}\Delta)-\exp(-\varphi_{i,n}\Delta)\right).$$

Eqn S5

Estimating expected numbers of infections

The effective reproductive number, $R_{i,n}$, is the expected number of infections host *i* would cause in the hosts remaining at time $t = T_n$, should it in fact be infected. In principle this could be calculated by multiplying the expected lifetime of the host by the rate at which it infects remaining susceptible hosts. The difficulty is that the lifetime of the host is difficult to estimate, depending in a complex fashion on the survey interval, the rate at which symptoms are expressed, and the culling strategy. However, since only relative values of $R_{i,n}$ are important (because estimates of risk are ranked against each other and then compared against a threshold parameter), we can arbitrarily use the survey interval Δ as the expected lifetime of an infected host, and set

$$R_{i,n} = \Delta\tilde{\beta} \sum_{\substack{j\in\{S(T_n),C(T_n)\}\\ j\neq i}} K(d_{ij};\tilde{\alpha}).$$

Eqn S6

Note that cryptically infected hosts are included in the estimate of $R_{i,n}$, since they would (erroneously) be assumed to be at risk of future infection.

Methods S2 Derivation of the variable radius control strategy.

The variable radius strategy is motivated by considering the implications of assuming each host is the only host to be infected. In particular, in deriving the method we assume only host *i* is infected and has been dispersing the pathogen for time Δ. Moreover, to allow the control around host *i* to depend entirely on its own reproductive number, we temporarily assume that all hosts within the neighbourhood of host *i* have the same basic reproductive number $R_{i,0}$. An estimate of the effective risk (cf Eqn 7 in the main text) for any other host *j* is then

$$E_j^{\text{eff}} = \left(1 - \exp(-\lambda\Delta)\right)\left[R_{i,0}\right]^{\gamma}, \qquad \text{Eqn S7}$$

in which

$$\lambda = \tilde{\beta} K(d_{ij}; \tilde{\alpha}), \qquad \text{Eqn S8}$$

is the force of infection from host *i* on host *j* at distance d_{ij} and the bias parameter is analogous to before. Assuming $\lambda\Delta$ is small, we can approximate the exponential term in Eqn S7 above, leading to

$$\begin{aligned} E_j^{\text{eff}} &= (1 - (1 - \lambda\Delta + \ldots))\left[R_{i,0}\right]^{\gamma}, \\ &\approx \lambda\Delta\left[R_{i,0}\right]^{\gamma}, \\ &= \tilde{\beta} K(d_{ij}; \tilde{\alpha})\Delta\left[R_{i,0}\right]^{\gamma}. \end{aligned} \qquad \text{Eqn S9}$$

By analogy with the risk-based strategy, the variable radius strategy then removes any host with effective risk above a pre-specified threshold value. This corresponds here to all hosts within a certain radius of the focal host, *i*, since the dispersal kernel is monotonically decreasing in distance. If the threshold effective risk were $E_{\text{thresh}}^{\text{eff}}$, then the maximum radius to cull around the ith host $R_i^{\max}$ would satisfy

$$E_{\text{thresh}}^{\text{eff}} = \tilde{\beta} K(R_i^{\max}; \tilde{\alpha}) \Delta \left[R_{i,0} \right]^{\gamma}. \qquad \text{Eqn S10}$$

We now define an adjustable parameter *R** as the control radius for a host with the average value of the reproductive number across the entire landscape, $\bar{R}_0$, where (cf Eqn 8)

$$\bar{R}_0 = \frac{1}{N} \sum_k R_{k,0} = \frac{1}{N} \sum_k \left(\Delta \tilde{\beta} \sum_{j \neq k} K(d_{jk}; \tilde{\alpha}) \right), \qquad \text{Eqn S11}$$

and where we have again used Δ as an estimate of the average infectious period (although both this and $\tilde{\beta}$ cancel out in Eqn S14 below, and so exact values are unimportant). This leads to

$$E_{\text{thresh}}^{\text{eff}} = \tilde{\beta} K(R^*; \tilde{\alpha}) \Delta \left[\bar{R}_0 \right]^{\gamma}. \qquad \text{Eqn S12}$$

Combining Eqns S10 and S12 gives

$$K(R^*; \tilde{\alpha}) \left[\bar{R}_0 \right]^{\gamma} = K(R_i^{\max}; \tilde{\alpha}) \left[R_{i,0} \right]^{\gamma}, \qquad \text{Eqn S13}$$

which is an implicit expression for $R_i^{\max}$ in terms of the dispersal kernel, the epidemiological parameters and the configurable bias and threshold parameters,   and *R**, respectively. Defining $\tilde{R}_{0,i}$, the basic reproductive number of the *i*th host relative to the average, as

$$\tilde{R}_{0,i} = \frac{\Delta \tilde{\beta} \sum_{j \neq i} K(d_{ij}; \tilde{\alpha})}{\frac{1}{N} \sum_k \Delta \tilde{\beta} \left(\sum_{j \neq k} K(d_{jk}; \tilde{\alpha}) \right)} = \frac{\sum_{j \neq i} K(d_{ij}; \tilde{\alpha})}{\frac{1}{N} \sum_k \left(\sum_{j \neq k} K(d_{jk}; \tilde{\alpha}) \right)}, \qquad \text{Eqn S14}$$

then Eqn S13 can be rewritten

$$K(R^*; \tilde{\alpha}) = K(R_i^{\max}; \tilde{\alpha}) \left[\tilde{R}_{i,0} \right]^{\gamma}. \qquad \text{Eqn S15}$$

For the Cauchy kernel, solving Eqn S15 for $R_i^{\max}$ (defaulting to a value of zero if this is impossible) leads to the following expression for the threshold radius

$$R_i^{\max} = \begin{cases} \tilde{\alpha}\sqrt{\left[\tilde{R}_{i,0}\right]^{\gamma}\left(1+\left(\frac{R^*}{\tilde{\alpha}}\right)^2\right)-1} & \text{if } \left[\tilde{R}_{i,0}\right]^{\gamma}\left(1+\left(\frac{R^*}{\tilde{\alpha}}\right)^2\right) > 1 \\ 0 & \text{otherwise} \end{cases}. \quad \text{Eqn S16}$$

For the exponential kernel the same procedure leads to

$$R_i^{\max} = \begin{cases} R^* + \gamma\alpha\log\left(\tilde{R}_{i,0}\right) & \text{if } R^* + \gamma\alpha\log\left(\tilde{R}_{i,0}\right) > 0 \\ 0 & \text{otherwise} \end{cases}, \quad \text{Eqn S17}$$

where in this case and if required $R_i^{\max}$ = 0 is adopted in favour of any biologically implausible negative values. In implementing the strategy we take $R_i^{\max}$ = 0 to correspond to simply roguing around host *i*, should it be detected as being infected.
